# Detection of *Leptomonas seymouri* narna-like virus in serum samples of visceral leishmaniasis patients and its possible role in disease pathogenesis

**DOI:** 10.1038/s41598-022-18526-9

**Published:** 2022-08-24

**Authors:** Soumi Sukla, Himadri Nath, Mohd. Kamran, Sarfaraz Ahmad Ejazi, Nahid Ali, Pradeep Das, V. Ravichandiran, Syamal Roy, Subhajit Biswas

**Affiliations:** 1 CSIR-Indian Institute of Chemical Biology, 4, Raja S.C. Mullick Road, Kolkata, West Bengal 700032 India; 2 National Institute of Pharmaceuticals Education and Research, 168, Maniktala Main Road, Kolkata, West Bengal 700054 India; 3 ICMR-Rajendra Memorial Research Institute of Medical Sciences, Agamkuan, Patna, Bihar 800007 India; 4 Academy of Scientific and Innovative Research (AcSIR), Ghaziabad, Uttar Pradesh 201002 India

**Keywords:** Clinical microbiology, Parasitology, Virology

## Abstract

Kala-azar/Visceral Leishmaniasis (VL) caused by *Leishmania donovani* (LD) is often associated with *Leptomonas seymouri* (LS) co-infection in India. *Leptomonas seymouri* narna-like virus 1 (Lepsey NLV1) has been reported in multi-passaged laboratory isolates of VL samples which showed LD-LS co-infection. A pertinent question was whether this virus of LS is detectable in direct clinical samples. DNA from the serum of twenty-eight LD diagnosed patients was subjected to LD-specific and LS-specific PCR to reconfirm the presence of LD parasites and to detect LD-LS co-infections. RNA extracted from same samples was subjected to RT-PCR, qRT-PCR and sequencing using virus-specific primers to detect/identify and quantify the virus. The presence of the virus was confirmed in thirteen of eighteen (72%) recently collected VL and PKDL samples. Cytokine profiling showed significantly elevated IL-18 in only LD infected patients compared to the virus-positive LD and control samples. IL-18 is crucial for Th1 and macrophage activation which eventually clears the parasite. The Lepsey NLV1 interaction with the immune system results in reduced IL-18 which favors LD survival and increased parasitic burden. The study emphasizes the need to revisit LD pathogenesis in the light of the association and persistence of a protozoan virus in kala-azar and PKDL patients.

## Introduction

Visceral leishmaniasis (VL) is one of the most important protozoan diseases caused by *Leishmania donovani* (LD). VL is fatal if left untreated. VL is commonly known as kala-azar in India. India is endemic for VL and sand flies (*Phlebotomus* spp) act as the insect vector. Some patients develop post-kala-azar dermal leishmaniasis (PKDL) whose pathogenesis is still considered an unresolved mystery^1^ and it is indicated that PKDL patients might act as silent parasite pool for transmission of leishmaniasis in kala-azar endemic areas^2^.

In South American countries, an aggravated form of cutaneous leishmaniasis (CL) has been attributed to be influenced by a protozoan dsRNA virus called Leishmania RNA virus1 (LRV1)^3,4^. LRV2 reported from old world leishmaniasis cases, showed a similar trend^5–7^. However, LRVs have never been reported from VL samples and despite rigorous attempts we too could not detect LRVs in the 22 well-characterized Indian VL cultured isolates^8^. Surprisingly, a different protozoan virus called *Leptomonas seymouri* narna-like virus 1 (Lepsey NLV1) was found in a great majority of these multi-passaged samples^8^. Lepsey NLV1 was not of LD but of *Leptomonas seymouri* (LS) origin. The latter (LS) is another protozoon transmitted by the sand fly vector of LD. Historically, LD clinical isolates from India often showed high incidence of LS co-infection^9–12^.

In our previous study, 20 out of the 22 (91%) LD isolates were LS-positive by PCR-based detection and 15 (75%) of the 20 LD-LS co-infected samples were Lepsey NLV1-positive. Ours was the first report that Indian VL/kala-azar victims are exposed to the LD-LS-Lepsey NLV1 triple pathogen complex^8^.

LD-LS coinfection of patients had been inferred from the analysis of cultured isolates in majority of the studies that have been published, including ours^8,9,11,12^. However, it is to be noted that this coinfection has also been demonstrated in lesional skin biopsy specimens from PKDL cases and in direct biopsy materials, namely peripheral blood from VL patients, at least on one occasion^10^. In the current study, we have screened for Lepsey NLV1 in archived and recently-collected human serum samples, from LD-diagnosed patients as well as endemic controls, rather than the promastigote stage of the parasites cultured in artificial growth media.

We have also analyzed the cytokine profile of serum samples of VL patients. IL-18 has been previously associated with LD infection and considered as important cytokine to elicit anti-LD adaptive immunity^13,14^. The serum levels of IL-18 for virus-positive and negative LD infected patients’ serum samples as well as endemic controls were assessed.

## Results

### Screening for Lepsey NLV1 in VL and PKDL samples

Screening for Lepsey NLV1 was conducted on recently collected (2017–2018; n = 18, 13VL and 5 PKDL cases) and archived (2014–2016; n = 10, all VL cases) serum samples of LD-diagnosed patients (Table 1).

**Table 1:** Screening of recent and archived Kala-azar/PKDL serum samples from India for Lepsey NLV1.

Sl. no	Sample	Year of collection	Recent (R)/Archived (A)	LD-PCR	LS-PCR	Lepsey NLV1 Semi-nested PCR	Virus RNA copies/ml serum
1	VL12	2014	A	+	−	+	8 × 10^6^
2	VL13	2014	A	+	−	−	−
3	VL14	2014	A	+	−	−	−
4	VL16	2014	A	+	−	−	−
5	VL17	2014	A	+	−	−	−
6	VL18	2015	A	+	+	f +	4 × 10^7^
7	VL15	2015	A	+	−	f +	2 × 10^7^
8	VL19	2015	A	+	−	+	1 × 10^7^
9	VL11	2016	A	+	−	−	−
10	VL20	2016	A	+	−	−	−
11	VL2	2017	R	+	+	+	5 × 10^6^
12	VL3	2017	R	+	+	+	3 × 10^6^
13	VL1	2017	R	+	−	+	4 × 10^6^
14	VL6	2017	R	+	−	+	3 × 10^6^
15	VL4	2017	R	+	−	f +	5 × 10^6^
16	VL21	2018	R	+	+	+	5 × 10^5^
17	VL10	2018	R	+	+	+	4 × 10^4^
18	VL5	2018	R	+	−	+	4 × 10^7^
19	VL22	2018	R	+	−	+	1 × 10^6^
20	VL8	2018	R	+	−	+	6 × 10^4^
21	VL9	2018	R	+	−	+	5 × 10^4^
22	VL7	2018	R	+	−	−	−
23	VL23	2018	R	+	−	−	−
24	PKDL1	2017	R	+	−	−	−
25	PKDL4	2018	R	+	−	+	1 × 10^5^
26	PKDL3	2018	R	+	−	+	6 × 10^4^
27	PKDL2	2018	R	+	−	−	−
28	PKDL5	2018	R	+	−	−	−
29–39	Endemic control (n = 11)	2010–2018	A/R	−	−	−	−

Among the recently collected samples, eleven of thirteen (85%) VL serum samples and two of five PKDL serum samples were virus-positive. Interestingly, four of ten (40%) archived VL serum samples were also virus-positive.

**Figure 1:**
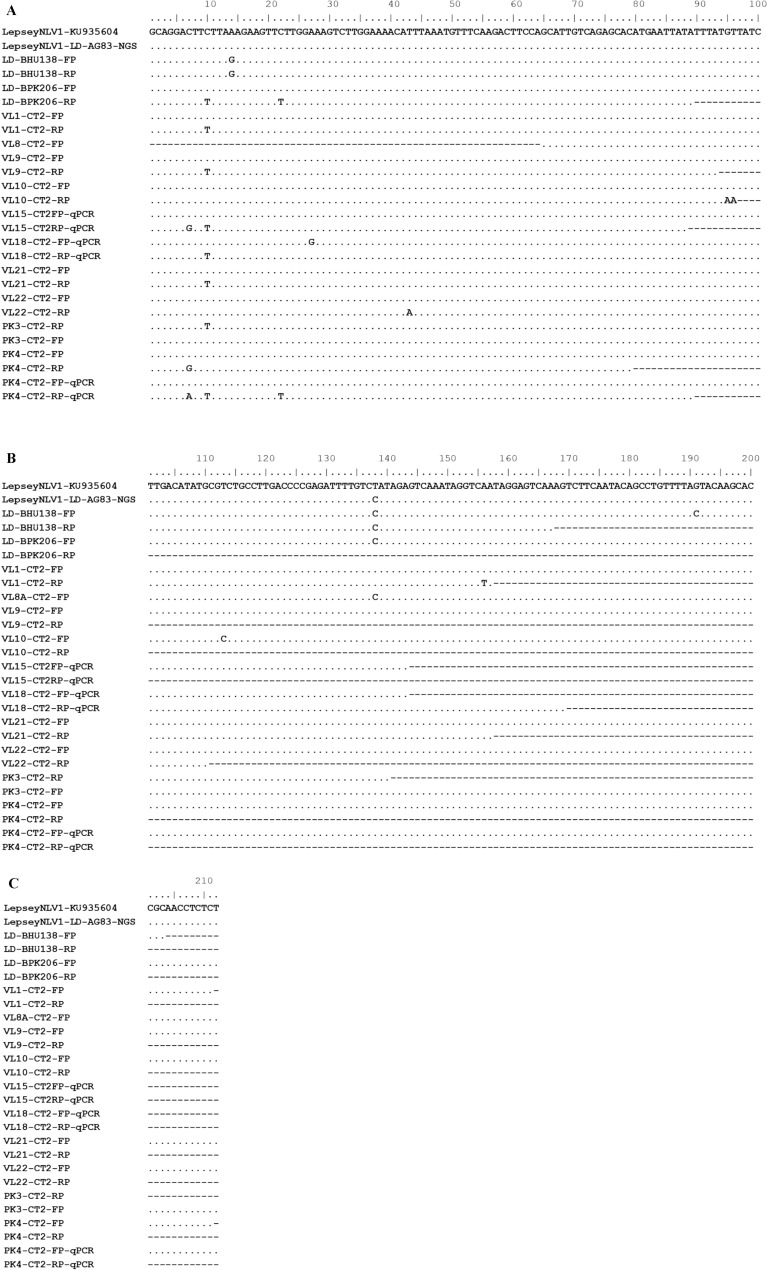
Representative L gene alignment (partial) of the Lepsey NLV1 detected in LD clinical samples. Partial segment L gene sequences of the Lepsey NLV1-positive LD samples and the Lepsey NLV1 isolate SBSS1 (obtained from AG83 by NGS sequencing; accession number KY628363) were aligned together with the only other Lepsey NLV1 sequence available in GenBank (accession number KU935604). The numbering of the nt positions corresponds to nt positions 167–378 of the Lepsey NLV1 sequence with accession number KU935604. FP and RP represent sequences obtained using relevant forward and reverse primers, respectively. “qPCR” suffix denotes that bidirectional sequencing was attempted on purified qPCR products. In the alignment, second to sixth sequences were from previously published virus-positive LD laboratory isolates^8^.

Overall, seventeen of twenty-eight LD samples (17/28 = 61%) showed evidence of the occurrence of the virus. The presence of the Lepsey NLV1 was confirmed by nested RT-PCR and qPCR on RNA extracted from the above-mentioned serum samples (n = 28) and DNA sequencing of the RT-PCR/qPCR products (Fig. 1). The estimated copy number of the viral genome ranged from 4 × 10^4^ to 4 × 10^7^ per ml of serum as determined by qPCR (Table 1).

The above-mentioned LD diagnosed samples (by LD-specific diagnostic tests) were reconfirmed of LD positivity by LD-specific PCR^8^. LS-specific PCR was also carried out and LS-DNA was detectable in only five of the twenty-eight serum samples from the aforesaid LD diagnosed patients (5/28 or 18%). All LD-LS positive samples were also virus-positive.

Serum samples from LD-negative and otherwise healthy individuals (n = 11) belonging to the endemic regions were found negative within the limits of threshold of the PCR used (data not shown). During screening, already known virus-positive and negative samples were used as respective controls for RNA extraction and RT-PCR.

### Cytokine array to check the profile of different cytokines

**Figure 2:**
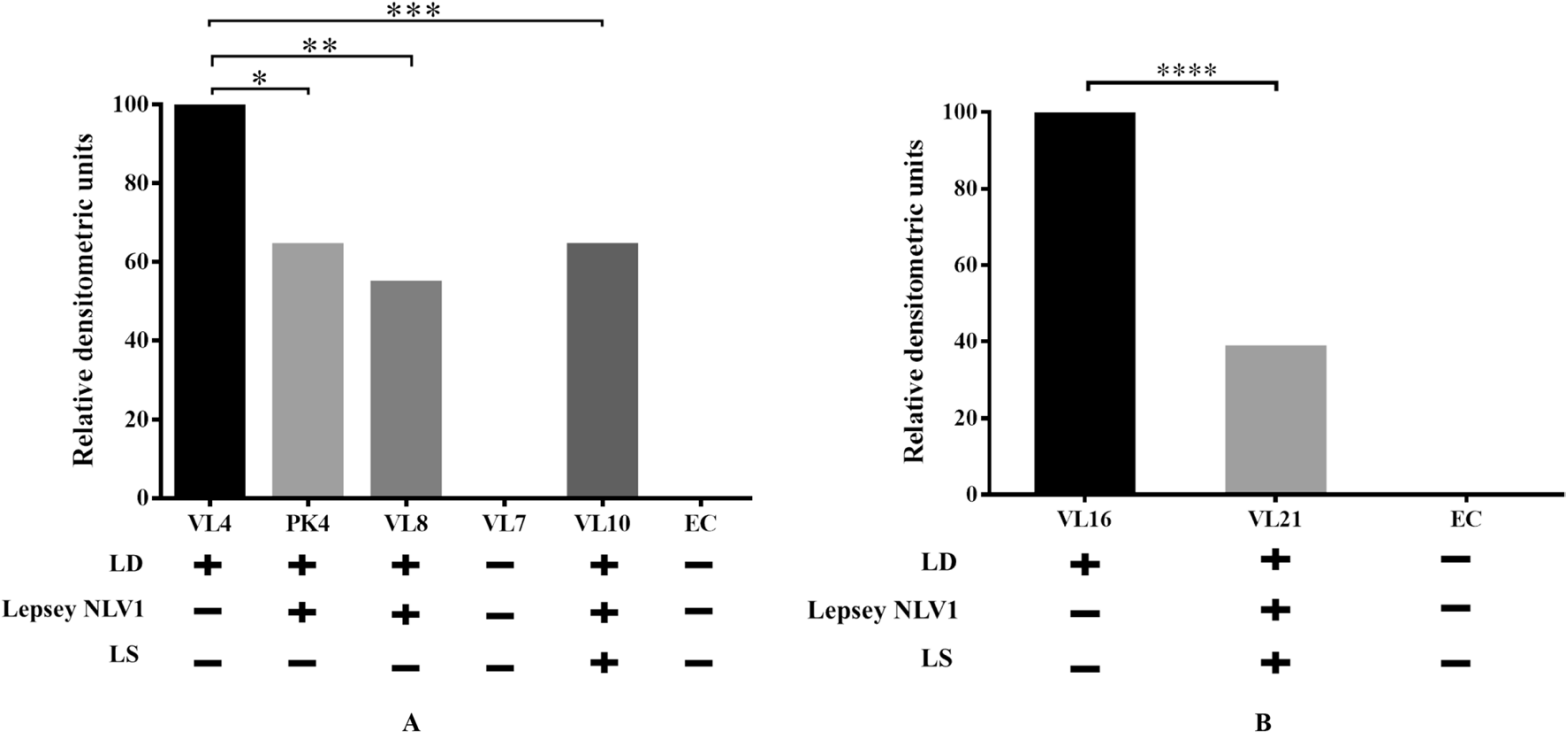
The levels of IL-18 in serum samples. Relative densitometric units as obtained in Proteome Profiler, were normalized in respect of only LD-containing samples. Samples were chosen based on LD, LS and Lepsey NLV1 test results. Sample VL7 was from a LD treated patient. “ + ” and “ − ” signs represent the presence and absence of three different microbes-*Leishmaina donovani* (LD), *Leptomonas seymouri* narna like virus 1 (Lepsey NLV1) and *Leptomonas seymouri* (LS). Panels A and B represent two different sets of experiments. EC-Endemic control. ‘*p*’ values were calculated from actual densitometric units observed from four replicates under each condition, by two tailed, unpaired t test at 95% level of significance. (*p = 0.0008, **p = 0.0005, ***p = 0.0007, ****p = 0.0002).

Serum samples were tested in cytokine array kit where a number of cytokines have been measured but the significant trend was observed only in case of IL-18. In endemic controls, IL-18 was below the detection threshold. In comparison with only LD infected patients, the coexistence of LD and Lepsey NLV1 resulted in statistically significant reduction in serum IL-18 level (Fig. 2). The VL7 sample was obtained from a LD treated person and IL-18 was not detected in it (Fig. 2, left panel). To check this trend of IL-18, quantitative ELISA was done using larger number of samples (Table 2).

**Table 2:** List of serum samples used in IL-18 ELISA.

LD (+); Lepsey NLV1 (−)	VL-treated	LD (+); Lepsey NLV1 (+)	PKDL-LD (+); Lepsey NLV1 (+)
VL13, VL14, VL16, VL17, VL11, VL20, VL23, PKDL1, PKDL2, PKDL5	VL7	VL12, VL18, VL15, VL19, VL2, VL3, VL1, VL6, VL4, VL21, VL10, VL5, VL22, VL8, VL9	PKDL4 PKDL3

**Figure 3:**
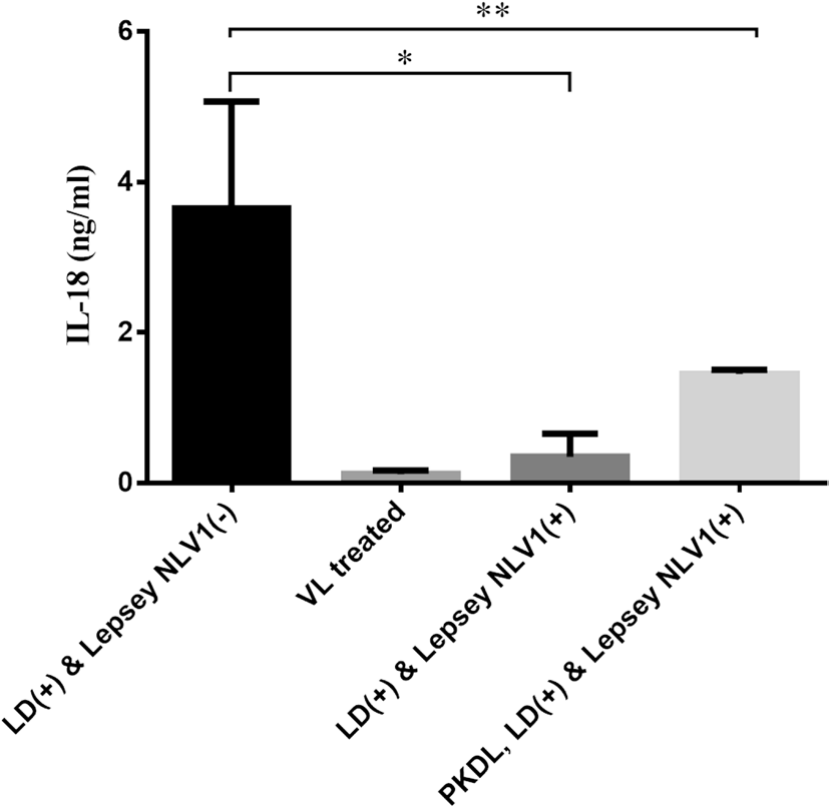
IL-18 in serum samples as found in quantitative IL-18 ELISA. Quantitative IL-18 ELISA was done as per manufacturer’s instructions. “(+)” and “(−)” signs represent the presence and absence of microbes-*Leishmaina donovani* (LD), *Leptomonas seymouri* narna like virus 1 (Lepsey NLV1). VL- Visceral Leishmania, PKDL-Post Kala-azar Dermal Leishmaniasis. ‘*p*’ values were calculated from actual IL-18 concentration in three replicates for each serum sample, by two tailed, unpaired *t* test at 95% level of significance. (*p = 0.002, **p = 0.0126).

The level of IL-18 was much higher in serum samples of patients infected with LD only, as revealed by the IL-18 quantitative ELISA (Fig. 3). LD only samples had significantly increased IL-18 than LD plus Lepsey NLV1 containing samples, supporting the results of the dot blot cytokine array. IL-18 level was much reduced in the VL-treated patient. The LD plus Lepsey NLV1 PKDL samples were also associated with significantly reduced IL-18 levels.

### Lepsey NLV1 genomic RNA is stable at 37 °C

Since majority of the serum samples were Lepsey NLV1-positive, including the archived samples, one obvious question was how the presumably naked RNA genome equivalents (gE) of Lepsey NLV1 could be detected for so long? This was tested and it was observed that the Lepsey NLV1 gEs were quite stable in serum. Only one log reduction was observed after 30 days of incubation at 37 °C (Table 3).

**Table 3:** Stability of Lepsey NLV1 genome at 37 °C.

Sample name	Lepsey NLV1 gEs/ml of serum			
	Given	Recovery after 7 days at 37 °C	Recovery after 15 days at 37 °C	Recovery after 30 days at 37 °C
VL12	8 × 10^6^	4.4 × 10^5^	3.7 × 10^5^	5.3 × 10^5^
VL18	4 × 10^7^	3.2 × 10^6^	2.7 × 10^6^	1.3 × 10^6^
VL15	2 × 10^7^	3.1 × 10^6^	2.4 × 10^6^	1.2 × 10^6^
VL19	1 × 10^7^	0.9 × 10^6^	0.7 × 10^6^	0.8 × 10^6^
VL2	5 × 10^6^	2.4 × 10^5^	3 × 10^5^	1.6 × 10^5^
VL3	3 × 10^6^	5.1 × 10^5^	6.4 × 10^5^	5.8 × 10^5^
VL1	4 × 10^6^	6.5 × 10^5^	5.4 × 10^5^	3.8 × 10^5^
VL6	3 × 10^6^	4.2 × 10^5^	1.7 × 10^5^	2.1 × 10^5^
VL5	4 × 10^7^	2.3 × 10^6^	3.3 × 10^6^	2.7 × 10^6^
VL22	1 × 10^6^	0.6 × 10^5^	0.4 × 10^6^	0.3 × 10^6^

## Discussion

It was observed that 61% of serum (recent and archived samples) from LD-diagnosed patients showed evidence of the presence of the virus by molecular diagnosis. Despite the fact that this virus is believed to be narna-like virus i.e. naked RNA-like virus, the RNA copy number estimated is relatively high (> 10^4^/ml serum). This holds true for even four years old archived samples (collected in 2014) and stored in − 80 °C freezer.

Only 18% of the LD-diagnosed serum samples (5/28, including one in the ten archived samples) were found LS-positive by PCR. In our previous study, it was observed that the LS-negative but LD-positive cultured isolates were also virus-negative by PCR^8^. Therefore, it appears that the LS originally present in the recent and archived clinical samples could not be detected in the serum by PCR due to their presumably low abundance compared to LD in majority of the co-infected virus-positive patients. Laboratory LD samples were passaged several times at 22 °C which possibly provided survival advantage to LS as the latter is known to grow faster at 22 °C^12^. Our aim in this study, was to check for the presence/absence/abundance of Lepsey NLV1 in direct human serum samples without giving any added advantage to LS parasites by artificial culturing at laboratory. So, there could be less LS parasites in the serum samples (considering the normal human body temperature i.e. 37 °C). LS has got survival disadvantage in the human system (37 °C) compared to the LD parasites but can grow relatively better in in vitro cultures with LD at 22–27 °C ^15^. This could be the reason why LS was beyond the detection level of the PCR test. There are reports stating high viral load (Lepsey NLV1) of LS parasites^7,12^ and this may contribute to detection of high Lepsey NLV1 RNA titer in LD-positive (and LS-positive but apparently PCR-non-detectable) patients' serum samples.

Detecting the RNA virus in 60% of the serum samples also indicates originally higher levels of LS co-infection than actually detected by PCR. This is possible because LS parasites have been reported to carry high load of the virus^12^, hence the virus could still be detected in apparently LS PCR-negative samples. We have attempted screening for Lepsey NLV1 in laboratory cultures of LS-negative LD clinical isolates but without any success so far (data not shown).

The detection of Lepsey NLV1 in apparently LS-negative serum samples raised an important question about the stability of virus/viral RNA at 37 °C. The viral RNA needs to be stable for at least a week to reflect its effect in host immunity^16^. In this context, Lepsey NLV1 genome has been observed to be quite stable in serum at 37 °C even after one month. So, the prolonged prevalence of Lepsey NLV1 genomes in LD patients’ serum samples should activate Toll-like Receptors (TLRs) and most likely to have an immunological relevance as well.

In context to the present report, it is noteworthy that LD-LS co-infection has been first time observed in unusual CL cases (38.5%) from Himachal Pradesh (northern India), recently. Whether these LD-LS co-infected samples contained Lepsey NLV1 is an open question^17^.

It has been well-documented that presence of LRV promotes LD persistence and exacerbates pathogenesis of leishmaniasis^3^. *Leishmania* spp infect and replicate in macrophages and the circulating macrophages are involved in the dissemination of the parasite in the body. LRVs found in South American *Leishmania* spp have been shown to bind to TLR3 and via type I IFN-mediated pathways, they inhibit ATG-5 mediated autophagy of NLRP3^18^ and IL-1β maturation^19^. Consequently, inflammasomes are inactivated and *Leishmania* replication within macrophages goes unabated. As a result of the above processes, *Leishmania* spp carrying the LRV, replicate more efficiently (compared to the virus-negative parasites) in macrophages and the infected macrophages survive better leading to parasite persistence^18–20^, dissemination/metastasis, hyper-pathogenesis^3^, parasite relapse and even drug-resistance^4^.

In this study, we have found high IL-18 in LD infected patients in comparison with endemic controls. It is quite expected as IL-18 is associated with IL-12 and IFN-γ production. IL-12 induces Th1 response; on the other hand, IFN- γ is required for macrophage activation. Both Th1 and macrophages are crucial for LD clearance^13^. Even for sodium stibogluconate (SSG) treatment, competent immune response with Th1 and macrophages, is necessary^13^. In support of this already established mechanism, we observed that IL-18 was not detected in a LD treated patient. We have also performed IFN-α ELISA of serum samples but in most of the cases it was below the detection limit (data not shown).

**Figure 4:**
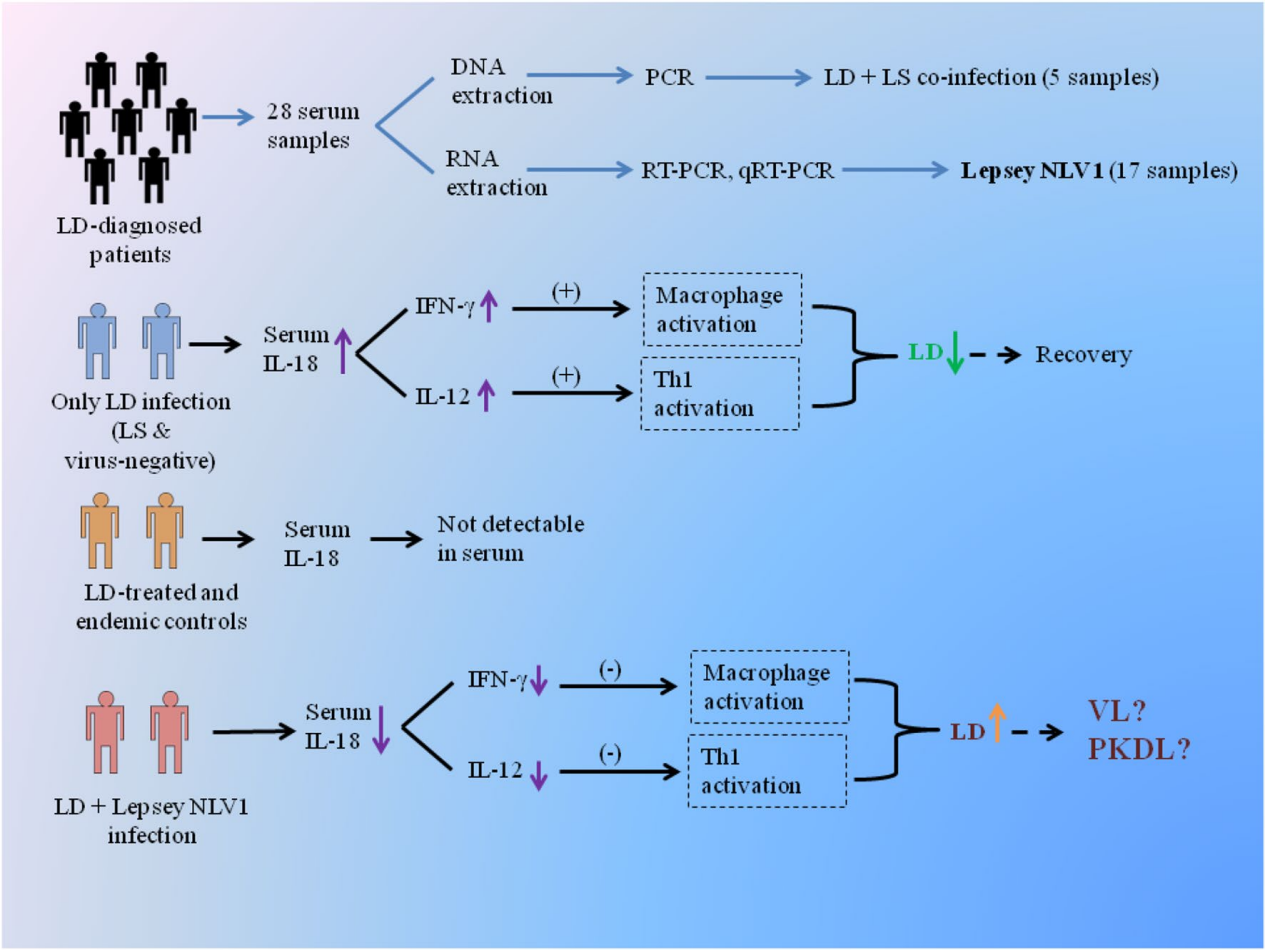
Schematic representation of putative role of Lepsey NLV1 in promoting persistence of LD. LD: *Leishmaina donovani*; LS: *Leptomonas seymouri*; Lepsey NLV1: *Leptomonas seymouri* narna like virus 1; Vertical up-arrow: up-regulated; Vertical down-arrow: down-regulated; (+): activation; (−): no activation.

However, in patients, where Lepsey NLV1 was present along with LD, the level of IL-18 in serum was consistently and significantly reduced compared to only LD infection. Studies have shown that IL-18 deficiency was associated with higher parasitic burden in later stage i.e. 40–44 days post-infection, in comparison with uninfected mice^13,14^. At that time point, IL-18 deficient mice had significantly lowered IL-12 and IFN-γ in the serum^13^ which justifies the increased LD burden. Now, as per our observation, it appears that Lepsey NLV1-interaction with the immune system (in presence of LD) down-regulates the IL-18 level in serum. This lowered IL-18 contributes to increased susceptibility to LD and subsequent persistence of LD. In the clinical scenario, VL patients have also been found to have reduced ability to produce IL-18^21^ which may be due to the presence of Lepsey NLV1. Although virus-positive LD clinical samples consistently showed lower IL-18 levels compared to the virus-negative LD clinical samples, we acknowledge that the number of samples in our study is not sufficient to come to any definitive conclusion. Further well-designed experiments are warranted to validate this preliminary observation (Fig. 4).

The role of protozoan viruses in Leishmaniasis pathogenesis and management is emerging as an important arena of research towards elucidating host versus multiple pathogens interaction^22–24^. We are currently pursuing questions such as how this protozoan virus persists in the human system for so long, and in considerable numbers, making its recovery possible from even archived human serum samples. It would be also important to investigate whether Lepsey NLV1 protozoan virus (detectable in high titers in human serum of LD-LS co-infected individuals), has any effect/influence on the patho-biogenesis of kala-azar (and PKDL). Lepsey NLV1 RNA being a Pathogen-associated molecular pattern or PAMP (like LRV dsRNAs), is expected to trigger TLR-induced type I IFN mediated pathways; further investigations in this direction are warranted. Visceral leishmaniasis remains the most neglected tropical disease in terms of drug discovery and availability of the drugs^25^ hence search for potent and affordable drugs are still a priority. It may be stated that the association and persistence of a protozoan virus in direct human clinical samples from kala-azar and PKDL patients re-emphasizes the need to revisit LD pathogenesis and management including treatment as part of the leishmaniasis surveillance program in India.

## Methods

### Ethical statement

The present study was approved by the respective Institutional Ethical and Biosafety Committees of CSIR-Indian Institute of Chemical Biology, Kolkata and RMRI, Patna. Written informed consents (in their native language) were obtained from all the patients/individuals before sample collection. All the experiments were carried out as per concerned guidelines and regulations.

### Subject of the study

Suspected patients from endemic regions were tested using rK39 strip test/splenic aspiration/bone marrow aspiration test or a combination of any two. Serum samples were obtained from LD-positive patients (n = 28). Details of the patients’ age, sex, and treatment status have been described in Table 4. Serum samples (n = 11) from otherwise healthy individuals (clinically diagnosed as LD-negative) from endemic areas, collected between 2010 and 2018 were also screened for the presence of the virus. Previously characterized and known virus-positive and negative LD isolates, cultured in vitro, were used as respective controls for RNA extraction and RT-PCR^8^.

**Table 4:** List of the serum samples along with the patient details.

Sl. no	Name of the samples	Age (in years)	Sex	Detection test (SA: Spleenic aspiration; BMA: Bone marrow aspiration)	Treatment status at the time of sample collection
1	**VL1**	4.5	M	rK39	Prior to treatment
2	**VL2**	45	M	BMA	Miltefosine (Mil) & Sodium antimony gluconate (SAG) treated
3	**VL3**	25	M	rK39, BMA	Prior to treatment
4	**VL4**	30	M	rK39	Prior to treatment
5	**VL5**	37	M	BMA	Mil & SAG treated
6	**VL6**	1.5	M	NA	Completed treatment
7	**VL7**	26	M	NA	Relapse
8	**VL8**	21	M	rK39, SA	Prior to treatment
9	**VL9**	24	M	rK39	Prior to treatment
10	**VL10**	25	M	rK39, SA	Prior to treatment
11	**VL11**	14	M	SA	Prior to treatment
12	**VL12**	45	M	BMA	Mil & SAG treated
13	**VL13**	24	M	rK39	Relapse, Mil treated
14	**VL14**	15	M	rK39	Prior to treatment
15	**VL15**	62	M	rK39, BMA	Completed treatment
16	**VL16**	60	F	rK39, SA	Prior to treatment
17	**VL17**	13	F	rK39	Prior to treatment
18	**VL18**	19	M	rK39	Prior to treatment
19	**VL19**	18	M	rK39	Prior to treatment
20	**VL20**	45	M	SA	Prior to treatment
21	**PKDL1**	16	M	rK39	PKDL cured
22	**PKDL2**	24	F	rK39	PKDL cured
23	**PKDL3**	19	M	rK39	PKDL cured
24	**PKDL4**	28	M	rK39	Prior to treatment
25	**VL21**	23	F	rK39	Prior to treatment
26	**VL22**	7	Not recorded	rK39	Prior to treatment
27	**VL23**	60	M	rK39	Prior to treatment
28	**PKDL5**	65	M	rK39	Prior to treatment

### Nucleic acid extraction and PCR amplification

DNA was extracted from serum and PCRs for LD and LS detection were performed following previously published protocol^8^. Total RNA from serum was extracted using the High Pure Viral Nucleic Acid Kit (Roche) following the manufacturer’s instructions. The concentration and purity of RNA were checked using spectrophotometry (Nanodrop One). cDNAs were prepared from total RNA using the Superscript III RT kit (Invitrogen) with the CT2-nRP reverse primer (5’- CGCATTCATTTGCGATCTCC-3’). Extracted cDNAs were then subjected to virus-specific nested PCR using GoTaq 2X Master Mix (Promega, USA).

Reaction for the first round PCR (665 bp product) comprised of GoTaq 2X Master Mix (25 µl), CT2-F and CT2-nRP primers (10 µM concentration; 1 µl each), template cDNA (10 µl) and nuclease-free water (13 µl) (Ambion). The second round hemi-nested PCR (338 bp) comprised of same ingredients as above except for the primers (CT2-F and CT2-R in this case; 10 µM concentration; 1 µl each). The template was 10 µl of tenfold diluted first round PCR product. The sequences for CT2-F and CT2-R primers are 5’-AACCCGAGGGTCAGTTTCTT-3’and 5’-TAAAGAGAGGTTGCGGTGCT-3’ respectively.

For each PCR, the cycling conditions were one cycle of initial denaturation at 95 °C (5 min); six cycles of touch-down PCR with annealing from 62 to 57 °C starting with denaturation at 94 °C (30 s), annealing at each descending temperature (30 s) and extension at 72 °C (1 min). This was followed by 40 cycles of denaturation at 94 °C (30 s); annealing at 56 °C (30 s) and extension at 72 °C (1 min). The PCR reaction concluded with final extension at 72 °C for 10 min followed by hold at 4 °C.

The PCR products were analysed on 1% agarose gels containing SYBR safe (Invitrogen) for nucleic acid staining. PCR bands of the correct size were either gel purified (QIAGEN Gel Extraction Kit, Germany) or PCR purified (QIAGEN PCR Purifcation Kit, Germany) prior to bi-directional DNA sequencing using CT2-F and CT2-R primers.

### Real-time PCR

A quantitative real-time PCR (qRT-PCR) was developed to determine the virus RNA copy number in the total nucleic acid extracted from the serum samples. A 338 bp fragment of the Lepsey NLV1 gene was amplified using the primers CT2-F and CT2-R primers.

A pcDNA3.1 (+) plasmid (Invitrogen, USA), containing the CT2-F and CT2-R PCR product cloned into it, was used as the standard for the real-time PCR. Serial dilutions of this recombinant plasmid were used as known standards to calculate the RNA copy number of the samples tested.

Each qPCR reaction (done in triplicate on each occasion) contained 10 µL of One-step Luna Universal qRT-PCR Master Mix (2X) (New England BioLabs), 1 µl RT, 0.5µL each of 10 µM forward and reverse primers, 2.5µL of standard or 1.0 µl (0.5 µg) RNA sample and RNase-free water in a final volume of 20 µL. The thermal cycling conditions in the Quant Studio 5 (Applied Biosystem) consisted of reverse transcription at 55 °C for 30 min; initial hold at 95 °C (1 min), followed by 40 cycles of 10 s at 95 °C, 30 s at 56 °C and extension at 72 °C (45 s). Fluorescence was monitored during this extension phase. The reaction finally concluded with a final extension step of 72 °C (5 min). Formation of bands of expected size was further confirmed by Agarose Gel Electrophoresis (AGEP) of qPCR products after completion of real-time PCR run. Visible bands from replicates were also purified and sequenced wherever possible.

### Proteome profiler cytokine array

Cytokine assay was performed as per manufacturer’s instruction using the R&D System Europe Ltd. Human Cytokine Array Kit (#ARY005B). Serum samples (200 µl) were incubated overnight with antibody-coated strips, provided in the Kit. The array strips were washed to remove unbound proteins. All the strips were then incubated with a cocktail of biotinylated detection antibodies along with streptavidin-HRP antibodies. Chemiluminescent signal was detected using the Azure Biosystems c400 imaging platform.

### IL-18 ELISA

Quantitative IL-18 ELISA was performed as per manufacturer’s instruction using Abcam Human IL-18 ELISA Kit (ab215539). Standard curve was prepared using dilutions of recombinant IL-18, provided in the kit.

### Checking the stability of Lepsey NLV1 RNA

Serum samples containing Lepsey NLV1 were kept in a 37 °C incubator up to 30 days. Each sample had three replicates, which were used for RNA extraction at three different time points, namely 7, 15 and 30 days post-incubation. Extracted RNA was quantified using the qRT-PCR, as mentioned previously.

### Ethics approval and consent to participate

The study was conducted according to the guidelines of the Declaration of Helsinki, and was approved by the respective Institutional Ethical and Biosafety Committees of CSIR-Indian Institute of Chemical Biology, Kolkata and ICMR- Rajendra Memorial Research Institute of Medical Sciences, Patna. Informed consent (in their native language) was obtained from all subjects involved in the study.

## Data Availability

Further information and requests for resources should be directed to and will be fulfilled by the corresponding authors, Dr Subhajit Biswas (subhajit.biswas@iicb.res.in) and Dr Soumi Sukla (soumi.sukla@niperkolkata.edu.in). All datasets presented in this study are included in the manuscript. Since the nucleotide sequence data are less than 200nt per sample, they could not be deposited in any public database. The sequences have been fully shown in the sequence alignment. Any additional data are available from the corresponding authors on request.
